# A genome-wide by PM_10_ exposure interaction study for blood pressure in Korean adults

**DOI:** 10.1038/s41598-023-40155-z

**Published:** 2023-08-11

**Authors:** Hyun-Jin Kim, Ho-young Son, Philiip Park, Jae Moon Yun, Hyuktae Kwon, Belong Cho, Jong-Il Kim, Jin-Ho Park

**Affiliations:** 1 National Cancer Control Institute, National Cancer Center, Goyang, South Korea; 2 Genomic Medicine Institute, Medical Research Center, Seoul National University, Seoul, South Korea; 3 Department of Family Medicine, Seoul National University Hospital, Seoul, South Korea; 4 Department of Family Medicine, Seoul National University College of Medicine, 103 Daehakro, Yeongun-Dong, Jongno-Gu, Seoul, 03080 South Korea; 5 Department of Biomedical Sciences, Seoul National University Graduate School, Seoul, South Korea; 6 Department of Biochemistry & Molecular Biology, Seoul National University College of Medicine, 103 Daehakro, Yeongun-Dong, Jongno-Gu, Seoul, 03080 South Korea; 7 Cancer Research Institute, Seoul National University College of Medicine, Seoul, South Korea

**Keywords:** Population genetics, Genetic association study, Genome-wide association studies, Genetic interaction

## Abstract

Blood pressure (BP) is a typical complex trait, and the genetic susceptibility of individuals to changes in BP induced by air pollution exposure is different. Although interactions of exposure to air pollutants with several candidate genes have been identified, genome-wide interaction studies (GWISs) are needed to understand the association between them with BP. Therefore, we aimed to discover the unique genetic loci for BP that interact with exposure to air pollutants in Korean adults. We ultimately included 1868 participants in the discovery step and classified them into groups of those with low-to-moderate exposure and high exposure to average annual concentration of particulate matter with an aerodynamic diameter ≤ 10 μm (PM_10_). Because none of the single nucleotide polymorphisms (SNPs) achieved a genome-wide level of significance of *p*_int_ < 5 × 10^–8^ for either systolic BP (SBP) or diastolic BP (DBP), we considered the top 10 ranking SNPs for each BP trait. To validate these suggestive SNPs, we finally selected six genetic variants for SBP and five variants for DBP, respectively. In a replication result for SBP, only one SNP (rs12914147) located in an intergenic region of the *NR2F2* showed a significant interaction. We also identified several genetic susceptibility loci (e.g., *CHST11*, *TEK*, and *ITGA1*) implicated in candidate mechanisms such as inflammation and oxidative stress in the discovery step, although their interaction effects were not replicated. Our study reports the first GWIS finding to our knowledge, and the association between exposure to PM_10_ and BP levels may be determined in part by several newly discovered genetic suggestive loci, including *NR2F2*.

## Introduction

Over the past decades, genome-wide association studies have led to remarkable achievements in understanding the genetic architecture of blood pressure (BP) regulation in humans^1,2^. However, BP is a classic complex trait that is influenced by multiple genetic and environmental factors, and it is also important to consider environmental factors^3^. In particular, exposure to ambient air pollution, including particulate matter (PM), is well known to be linked to increased BP in several epidemiological studies^4–7^. However, even if exposed to the same concentration of ambient air pollution, genetic susceptibility to BP increments varies between individuals, which may be explained by interactions between genetic and environment components, at least in part.

The biological pathology underlying the relationship between air pollution and BP level is not yet elucidated, but several candidate hypotheses have been proposed, such as inflammatory and oxidative stress responses^8,9^. Previous studies of genetic interaction with air pollution have been mostly conducted with limited numbers of candidate genes (e.g., *GSTM1*, *GSTT1*, and *GSTP1*) involved in the mechanisms^10,11^. Recently, our research team conducted an interactive genetic study focusing on the interesting relationship between obesity, air pollution, and elevated BP. We discovered new biological evidence that the genetic variants of *CDH13*, known as a susceptibility locus for adiponectin levels, enhance the association of exposure to PM with an aerodynamic diameter ≤ 10 μm (PM_10_) with increased BP^12^. In this context, it is important to elucidate additional evidence for an individual’s genetic susceptibility to changes in BP due to air pollution.

Genome-wide interaction studies (GWISs), which test genetic variants across the entire genome simultaneously, without any prior hypothesis such as biological theory, are a useful approach for discovering interactive genetic loci. New findings might help us to understand better the genetic background of key mechanisms that explain the association of increased BP with air pollution. However, to our knowledge, the interaction between air pollution and BP has not been studied on a genome-wide scale. Therefore, we conducted a GWIS of the association of PM_10_ exposure with BP traits in Korean adults and report the results across the entire genome.

## Results

### Population characteristics

Descriptive characteristics of the participants included in the discovery and replication steps are shown in Table 1. In the discovery step, all participants were adult men aged 21–68 years (n for recruitment A = 1355 and n for recruitment B = 513, respectively). The mean BMI, DBP, and use of antihypertensive medication were similar between the Health Promotion Center of Seoul National University Hospital (north of Seoul, site A) and Healthcare System Gangnam Center (south of Seoul, site B). The mean SBP and exposure to PM_10_ were different between the sites. In the replication step, a total of 1281 samples from adult participants were used. The proportion of women was 44.18% (n = 327) and their mean age was 54.36 (20–83 years). In the case of men in the replication step, the mean (SD) BMI was 24.31 (3.03) kg/m^2^, SBP was 126.87 (14.65) mmHg, and DBP was 77.28 (9.68) mmHg, and were similar to those values at discovery site A (data not shown).

**Table 1:** Basic characteristics of study subjects.

Characteristics	Discovery step		Replication step
	n (%) or mean (SD)		n (%) or mean (SD)
Year of sample recruitment	‘09–‘13		‘14–‘15
Site	A	B	A
N	1355	513	1281
Age years	49.06 (7.13)	48.51 (6.52)	54.36 (11.43)
Sex			
Men	1355 (100%)	513 (100%)	715 (55.82%)
Women	–	–	566 (44.18%)
BMI (kg/m^2^)	24.62 (2.78)	24.72 (2.77)	23.47 (3.15)
Blood pressure			
SBP (mmHg)	126.90 (13.91)	118.07 (12.94)	123.83 (15.63)
DBP (mmHg)	78.04 (10.44)	79.16 (10.71)	75.06 (10.08)
Antihypertensive medication			
Yes	255 (18.92%)	88 (17.15%)	338 (26.38%)
No	1099 (81.11%)	425 (82.85%)	942 (73.54%)
Missing	1 (0.07%)	–	1 (0.08%)
Glucose (mg/dL)	93.96 (18.59)	97.13 (17.55)	96.94 (20.96)
eGFR (mL/min per 1.73 m^2^)	81.21 (12.80)	77.95 (10.32)	93.11 (16.40)
Uric-acid (mg/dL)	6.38 (1.33)	6.41 (1.26)	5.65 (1.39)
Total cholesterol (mg/dL)	200.12 (35.16)	201.80 (36.65)	198.77 (39.53)
PM_10_ (μg/m^3^)			
Mean	47.96 (7.23)	52.71 (6.70)	47.79 (5.79)
25th percentile	42.94	48.86	44.43
50th percentile (median)	47.43	52.36	46.90
75th percentile	52.15	54.74	50.65

*BMI* body mass index, *SBP* systolic blood pressure, *DBP* diastolic blood pressure, *eGFR* estimated glomerular filtration rate, *PM*_*10*_ particulate matter with aerodynamic diameter ≤ 10 μm, *SD* standard deviation.

### Genome-wide interaction study

**Figure. 1:**
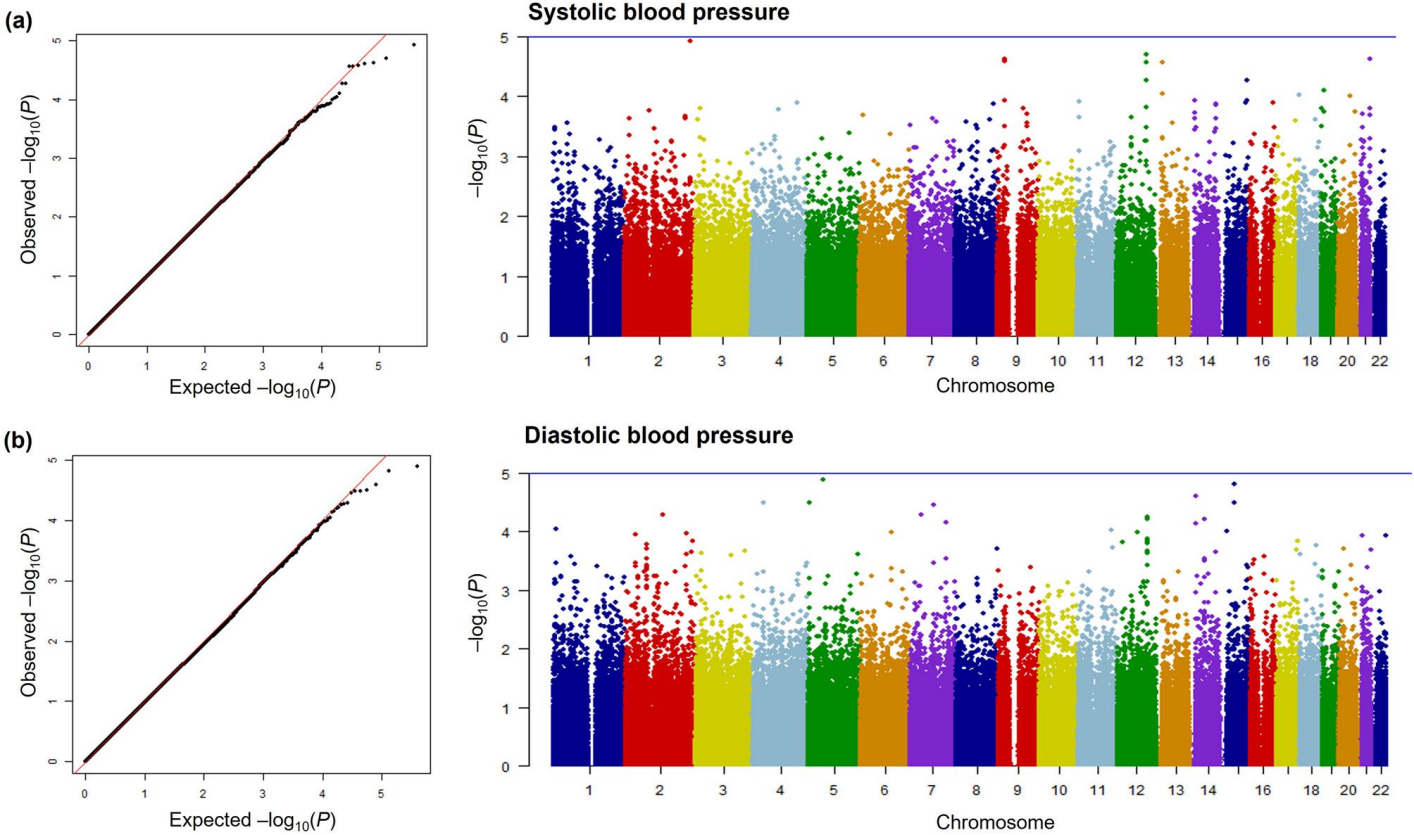
Genome-wide interaction plots for blood pressure. Quantile–quantile plots and Manhattan plots for (**a**) systolic blood pressure and (**b**) diastolic blood pressure.

The results of the genome-wide interaction analysis of the association of exposure to PM_10_ with SBP and DBP are shown in Fig. 1 and Table 2. Figure 1 depicts quantile–quantile (Q–Q) and Manhattan plots of *p*_int_ from the GWIS using 196,963 SNPs. A Q–Q plot showed little evidence for genomic statistic inflation. The top 10 genome-wide interaction results for the association of PM_10_ exposure with each BP parameter are summarized in Table 2. For SBP, the top SNP (rs13402716, *p*_int_ = 1.20 × 10^–5^) was observed near *SPHKAP* on chromosome 2. The second associated SNP (rs1795849, *p*_int_ = 1.99 × 10^–5^) and two other SNPs (rs10861229, *p*_int_ = 2.78 × 10^–5^, and rs10778338, *p*_int_ = 5.42 × 10^–5^) were located in the intron of *CHST11* and shared a high LD (*r*^2^ > 0.8). We also identified two SNPs (rs2273717 and rs1555454) located in the intron region of *TEK*, and the correlation between them was strong (*r*^2^ > 0.8). In addition, an interaction of rs12914147, which was located 98 kb away from *NR2F2*, with PM_10_ exposure associated with SBP was observed (*p*_int_ = 5.44 × 10^–5^). The SNP most associated with DBP (rs9686276, *p*_int_ = 1.29 × 10^–5^) was located in an intron of *ITGA1* on chromosome 5. The SNP second most associated with DBP (rs8033729, *p*_int_ = 1.54 × 10^–5^) and another SNP (rs2965318, *p*_int_ = 3.24 × 10^–5^) were located nearby *LINC01491* and shared a weak LD (*r*^2^ = 0.46).

**Table 2:** Genome-wide interaction results with PM_10_ exposure (exposed and non-exposed groups) for blood pressure (Top 10 SNPs).

Trait	Chr	SNP	Position^a^	Major/minor allele	MAF	Type	Nearest genes (distance, kb)	Model 1^d^		Model 2^e^	
								*β (SE)*	*P*_*int*_	*β (SE)*	*P*_*int*_
SBP	2	rs13402716	229331340	A/G	0.108	Intergenic	*SPHKAP* (284.9)	0.026 (0.006)	1.20E−05	0.023 (0.006)	5.27E−05
	12	rs1795849^b^	104911246	T/C	0.332	Intron	*CHST11* (0)	0.016 (0.004)	1.99E−05	0.015 (0.004)	4.50E−05
	9	rs2273717^c^	27228022	G/A	0.266	Intron	*TEK* (0)	0.017 (0.004)	2.40E−05	0.017 (0.004)	2.17E−05
	21	rs2838433	45333865	A/G	0.216	Intron	*AGPAT3* (0)	− 0.019 (0.005)	2.46E−05	− 0.019 (0.005)	4.63E−05
	9	rs1555454^c^	27228754	T/C	0.270	Intron	*TEK* (0)	0.017 (0.004)	2.68E−05	0.017 (0.004)	2.60E−05
	13	rs12585154	25204157	G/T	0.214	Intergenic	*TPTE2P6* (32.3)	− 0.019 (0.004)	2.76E−05	− 0.018 (0.004)	4.16E−05
	12	rs10861229^b^	104906745	G/T	0.342	Intron	*CHST11* (0)	0.016 (0.004)	2.78E−05	0.015 (0.004)	7.05E−05
	12	rs10778338^b^	104894402	A/G	0.342	Intron	*CHST11* (0)	0.016 (0.004)	5.42E−05	0.015 (0.004)	1.32E−04
	15	rs12914147	96981538	T/C	0.339	Intergenic	*NR2F2* (98.0)	− 0.016 (0.004)	5.44E−05	− 0.015 (0.004)	8.60E−05
	19	rs317913	11510314	A/G	0.276	Intron	*RGL3* (0)	− 0.017 (0.004)	7.97E−05	− 0.018 (0.004)	1.69E−05
DBP	5	rs9686276	52226067	C/A	0.339	Intron	*ITGA1* (0)	0.203 (0.046)	1.29E−05	0.192 (0.046)	3.29E−05
	15	rs8033729	48270609	A/G	0.419	Intergenic	*LINC01491* (132.2)	0.197 (0.046)	1.54E−05	0.195 (0.045)	1.65E−05
	14	rs12437363	20694079	A/G	0.173	Intergenic	*OR11H6* (1.2)	0.252 (0.060)	2.57E−05	0.252 (0.059)	2.01E−05
	4	rs10856831	37427661	G/A	0.200	Intron	*NWD2* (0)	0.231 (0.056)	3.21E−05	0.221 (0.055)	6.32E−05
	15	rs2965318	48264495	G/T	0.411	Intergenic	*LINC01491* (126.1)	− 0.190 (0.046)	3.24E−05	− 0.189 (0.045)	3.04E−05
	5	rs7714315	2818295	C/T	0.241	Intergenic	*C5orf38* (62.8)	− 0.217 (0.052)	3.25E−05	− 0.21 (0.052)	4.69E−05
	7	rs258723	81772396	A/G	0.471	Intron	*CACNA2D1* (0)	− 0.194 (0.047)	3.51E−05	− 0.186 (0.046)	6.38E−05
	2	rs841470	129370246	T/C	0.105	Intergenic	*LOC101927881* (251.9)	− 0.310 (0.076)	5.17E−05	− 0.293 (0.076)	1.13E−04
	7	rs1235304	38979827	C/T	0.480	Intergenic	*VPS41* (31.0)	0.186 (0.046)	5.34E−05	0.179 (0.045)	8.89E−05
	12	rs1882492^b^	104888494	G/A	0.392	Intron	*CHST11* (0)	0.182 (0.045)	5.59E−05	0.169 (0.045)	1.58E−04

*SBP* systolic blood pressure, *DBP* diastolic blood pressure, *Chr* chromosome, *SNP* single nucleotide polymorphism, *MAF* minor allele frequency.

^a^SNP positions are based on Human GRCh37/hg19 from UCSC Genome Browser.

^b,c^These SNPs were in the strong linkage disequilibrium (LD) relationship (r^2^ > 0.8; D′ = 1).

^d^Model 1: Adjusted for age, center, BMI.

^e^Model 2: Further adjusted for glucose, eGFR, uric-acid, total cholesterol.

We conducted additional interaction test with another adjustment models (model 1: original adjustment, model 2: further adjusted for glucose, estimated glomerular filtration rate (eGFR), uric-acid, total cholesterol, Table 2). We observed that interaction P-value and effect were not significantly different according to each adjustment models.

### Replication study

**Figure. 2:**
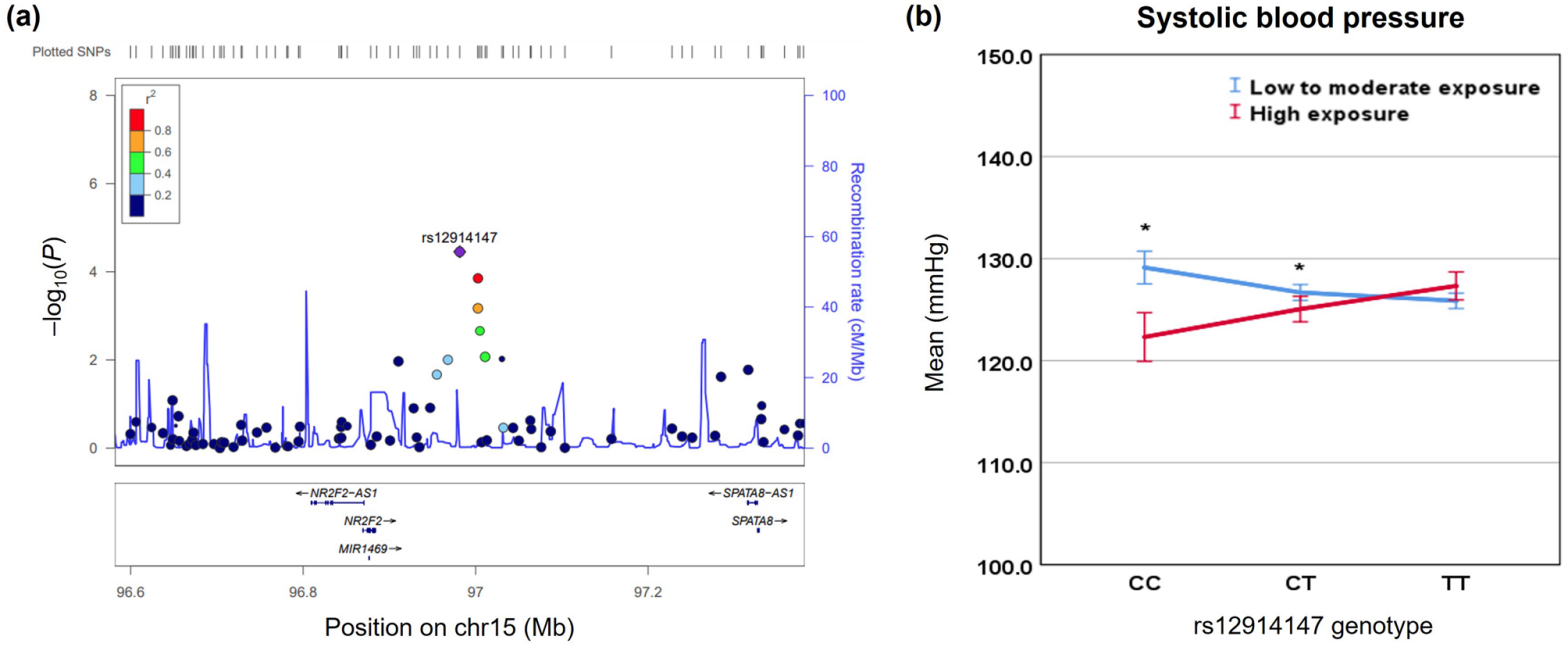
Regional association and interaction plots in *NR2F2* locus. (**a**) Regional association plots for *NR2F2* gene and (**b**) the interaction of rs12914147 genotype and PM_10_ exposure in modulating systolic blood pressure level (*p < 0.05).

To validate the GWIS result from the discovery step, we conducted a replication study with another 1,281 Korean adult participants (Table 1). Among the top 10 candidate SNPs for each BP interaction result, six SNPs for SBP and five SNPs for DBP were selected for the replication study, based on the priority of gene function and LD. Table 3 presents the interaction results from the replication sample. We identified that only one SNP (rs12914147) near *NR2F2* for SBP was replicated at a significance level of 0.05 (*p*_int_ = 0.034). We also conducted an association analysis for two groups stratified with non- or one minor allele (TT or TC genotype) and the homozygous carriers of minor allele (CC genotype) (Table 3). The PM_10_ exposure in the homozygous carriers of minor allele of rs12914147 was significantly associated with a decreased level of SBP (*p*_assoc_ = 0.019). By contrast, PM_10_ exposure in the group with no minor allele or one minor allele (TT or TC genotype) of rs12914147 did not show any significant association with SBP (*p*_assoc_ = 0.664). Detailed regional information near *NR2F2* and the interaction of the rs12914147 and PM_10_ exposure in modulating BP levels is shown in Fig. 2.

**Table 3:** Interaction results with PM_10_ exposure (exposed and non-exposed groups) for blood pressure in replication sample (n = 1281).

Trait	Chr	SNP	Gene	Non or one minor allele group		Homozygous carriers of minor allele group		Model 1^a^	Model 2^b^
				*β*_*assoc*_ (SE)	*P*_*assoc*_	*β*_*assoc*_ (SE)	*P*_*assoc*_	*P*_*int*_	*P*_*int*_
SBP	9	rs2273717	*TEK*	− 0.156 (0.235)	0.506	1.284 (1.088)	0.242	0.276	0.281
	12	rs1795849	*CHST11*	− 0.123 (0.241)	0.610	0.045 (0.777)	0.954	0.791	0.723
	13	rs12585154	*TPTE2P6*	− 0.098 (0.237)	0.679	0.723 (1.119)	0.521	0.424	0.362
	**15**	**rs12914147**	***NR2F2***	**0.104 (0.240)**	**0.664**	− **1.813 (0.762)**	**0.019**	**0.034**	**0.048**
	19	rs317913	*RGL3*	− 0.127 (0.239)	0.593	0.612 (0.728)	0.403	0.386	0.378
	21	rs2838433	*AGPAT3*	− 0.112 (0.237)	0.636	0.762 (1.038)	0.466	0.401	0.383
DBP	4	rs10856831	*NWD2*	− 0.011 (0.022)	0.613	− 0.100 (0.120)	0.410	0.562	0.281
	5	rs7714315	*C5orf38*	− 0.008 (0.022)	0.721	− 0.057 (0.096)	0.556	0.604	0.516
	5	rs9686276	*ITGA1*	− 0.013 (0.023)	0.573	− 0.055 (0.063)	0.389	0.683	0.732
	7	rs258723	*CACNA2D1*	0.000 (0.024)	0.984	− 0.047 (0.045)	0.302	0.381	0.364
	14	rs12437363	*OR11H6*	− 0.016 (0.022)	0.468	0.063 (0.141)	0.659	0.609	0.686

*SBP* systolic blood pressure, *DBP* diastolic blood pressure, *Chr* chromosome, *SNP* single nucleotide polymorphism, *SE* standard error.

Nominal association is marked in bold (*P*_int_ < 0.05).

^a^Model 1: Adjusted for age, sex, BMI.

^b^Model 2: Further adjusted for glucose, eGFR, uric-acid, total cholesterol.

We also conducted additional interaction test with another adjustment models in replication step (Table 3). Similar to the discovery step, we observed that the interaction was not significantly different.

## Discussion

The present study aimed to identify genetic susceptibility loci that are associated with increased BP levels through their interaction with PM_10_ exposure in an Asian population. We performed a GWIS of the association of exposure to PM_10_ with BP traits, such as SBP and DBP, in Korean adults. For both BP traits, none of the SNPs reached a stringent threshold for genome-wide significance (*p*_int_ < 5 × 10^–8^). Therefore, we present the top 10 candidate SNPs for each trait. Of these SNPs, six suggestive SNPs for SBP and five for DBP were ultimately included in the replication analysis step, considering the distance with nearest genes and the LD relationship between the SNPs. Only one SNP (rs12914147) for SBP was replicated at a nominal significance level of 0.05. This variant, located near *NR2F2*, was associated with a decreased level of SBP through its interaction with PM_10_ exposure. It remains "curious" to observe how exposure to PM_10_ is associated with lower systolic blood pressure values in CC homozygotes, while exposure to PM_10_ is usually considered a risk factor for hypertension. Nevertheless, this finding may emphasize the different genetic susceptibility to PM_10_ exposure and the related genetic-environmental interactions. At the discovery step, we also observed several suggestive genetic loci involved in biologically plausible mechanisms, such as inflammatory responses, even though their interaction effects were not replicated. Our findings highlight the importance of a genome-wide approach to discover genetic loci as well as considering genetic components to understand better the link between exposure to air pollution and BP levels.

Nuclear receptor subfamily 2 group F member 2 (*NR2F2*), also known as COUP-TF II, is a member of the nuclear orphan receptor superfamily. It is widely expressed in multiple tissues, including the lung, kidney, liver, spleen, and skeletal muscle. In 1999, Pereira et al. demonstrated that *NR2F2* plays an important role in angiogenesis and heart development^13^. *NR2F2* is also related to the regeneration of the pulmonary vascular endothelium after injury from influenza^14^. In addition, Lin et al. reported that the expression of *NR2F2* is mediated by proinflammatory cytokines including TNF-α, IL-1β, and TGF-β1^15^. Moreover, the gene for *NR2F2* is expressed in adipose tissue in vivo and is a potent suppressor in the regulation of adipogenesis^16^. Similarly, *NR2F2* is implicated in various mechanisms closely related to BP. In 2014, an exome-sequencing study identified that several rare variants within the coding region of *NR2F2* contribute to congenital heart defects in humans^17^. The possibility that *NR2F2* is linked to BP levels has also been shown in several genome-wide linkage studies of humans^18,19^. Furthermore, BP levels in Nr2f2^mutant^ rats were significantly lower than in controls^20^. Interestingly, the biological functions of *NR2F2* mentioned above, such as its role in the inflammatory response, are also involved in the key mechanisms linking air pollution and BP levels; the interaction between *NR2F2* and PM_10_ exposure may be due to a mechanism shared between them.

In addition to *NR2F2*, we identified several other potentially interacting loci (e.g., *CHST11*, *TEK*, and *ITGA1*) involved in plausibly associated biological pathways such as oxidative stress and inflammation responses, although their signals were not replicated in independent samples. *CHST11* belongs to the sulfotransferase 2 family and is associated with *TGF-β1*-induced production of reactive oxygen species (ROS) in human vascular smooth muscle cells^21^. The oxidative stress caused by inducing ROS production plays an important role in the pathogenesis of elevated BP^22^. We also found *TEK* as a locus potentially interacting with PM_10_ exposure. *TEK*, a member of the tyrosine kinase Tie2 family, not only regulates angiogenesis but also promotes anti-inflammatory responses^23^. Tie2 signaling is a crucial angiogenic mediator between the proinflammatory cytokine *TNF-α* and pathological angiogenesis in rheumatoid arthritis^24^. Finally, *ITGA1*, which encodes the α1 subunit of integrin receptors, plays a role in macrophage exit from peripheral inflammatory lesions^25^. *ITGA1* is upregulated in lung tissue of patients with pulmonary hypertension^26^. *CHST11*, *TEK*, and *ITGA1* and prolonged exposure to air pollution are associated with inflammatory or ROS responses, thereby leading to increased BP.

To date, gene-by-air pollution interactions with BP have been only identified in specific genes involved in miRNA processing, oxidative stress pathways, or etiology of the disease^10–12,27,28^. Wilker et al. found that the PM_2.5_-associated BP change is modified by candidate genes related to chronic obstructive pulmonary disease and asthma. They also observed an interaction of 7-day black carbon exposure and genetic variants in miRNA processing genes with BP^28^. Levinsson et al. investigated whether genetic polymorphisms in genes related to oxidative stress, such as *GSTT1*, *GSTP1*, and *GSTCD*, modify the association between long-term exposure to traffic-related air pollution and hypertension. They found that three *GSTP1* SNPs showed a significant association with hypertension, but no obvious interaction effect with air pollution exposure was observed^11^. However, a study of elderly Koreans identified that air pollution exposure associated with BP was modified by genetic risk scores generated using SNPs in a gene related to oxidative stress and suggested that an oxidative stress pathway may be an important link between air pollution and BP^10^. In addition, Kim et al. conducted a study of interactions of *CDH13* polymorphisms-by-PM_10_ exposure and reported that the variants increased susceptibility of BP increase to PM_10_ exposure^12^. Despite the discovery of these candidate genes, additional research on the entire genome without any prior information or hypothesis is needed to identify new candidate genetic loci that interact with air pollution.

To our knowledge, the present study is the first GWIS of the interaction of SNPs with PM_10_ exposure in relation to BP. Our new findings of several suggestive genes (e.g., *NR2F2*, *CHST11*, *TEK*, and *ITGA1*) that interact with air pollutant exposure may be important to understand better additional genetic and biological background for the association between air pollution and BP in the general adult population. Nevertheless, our study has some limitations that need to be considered. First, we could not accurately estimate air pollution exposure at an individual level because of missing relevant information such as the level of exposure indoors or at the workplace and how close participant’s houses are to major roads. Thus, the method of estimation using zip codes alone may misclassify individual exposure levels. Second, no SNPs reached a genome-wide significance for interaction (*p*_int_ < 5 × 10^–8^). This cut-off corresponds to the Bonferroni correction that assumes independence among 1 million SNP markers, which may be too conservative given the relationship between correlated SNPs. Thus, we considered the top 10 ranking SNPs for each BP trait. Similarly, because none of the candidate SNPs for replication achieved a stringent significance level of [*p*_int_ < 0.05/11 (#SNP tested) = 0.0045], we applied a nominal threshold of *p*_int_ < 0.05. This may be due to the small sample size of men (n = 715) in the replicate set. Anyway, it should be taken into account that these modified and less stringent threshold values could lead to a substantial risk of false discovery rate. Third, BP was evaluated with a single measurement. It is known that it’s better to measure the average of two or more BP measurements, and the one times measurement tends to be rather high result^29^. Nevertheless, we strictly followed the main requirements for BP measurement^30^ and tried to control other factors that could lead to exaggerated BP measurements (e.g., resting relaxed state, fasting state-no caffein/smoking with empty bladder-comfortable environment in the examination center, not in front of a doctor). Thus, we believe the overall overestimation probability is not large and it will not affect the result. Fourth, the sample in the discovery group was comprised entirely of adult male participants, and we may not be able to generalize findings to women and girls. Finally, exposure to PM_2.5_ could not be adjusted for in our interaction analysis, due to the lack of relevant data.

We evaluated the genome-wide interaction of SNPs with PM_10_ exposure associated with BP and found a suggestive genetic variant near *NR2F2*. We identified several possible loci (e.g., *CHST11*, *TEK*, and *ITGA1*) that play roles in plausible biological mechanisms such as inflammation or oxidative stress. These findings provide a new basis to understand better the additional genetic background beyond previously known genes in the association between air pollution and BP levels. Not only are replication studies needed to validate these candidate loci in a large sample and other populations, but further research at the genome-wide level is needed to discover more candidate loci.

## Methods

### Study participants

We conducted a cross-sectional study designed to evaluate the association between genetic variants and health outcomes such as anthropometric measurement, visceral fat, and metabolic traits. The participants in the present study visited the Health Promotion Center and Healthcare System Gangnam Center at Seoul National University Hospital from December 2009 to December 2013. The detailed recruitment methods have been described in our previous study^31^. Briefly, we enrolled 2102 participants and excluded 276 individuals for whom required information, such as BP-related phenotypes, a qualified DNA sample, and zip-code data to estimate the level of exposure to ambient PM_10_ concentration, was missing. We ultimately included 1868 adult men in the present genetic analysis. The Institutional Review Board of the Seoul National University Hospital Biomedical Research Institute and the National Cancer Center approved the study. Informed consent was obtained from all participants. All of the clinical investigations were conducted accordance with the Declaration of Helsinki.

### Assessment of phenotype measurement

BP was measured with participants sitting at rest for at least 5 min. For individuals who were taking antihypertensive drugs, we added 10 mmHg to the observed SBP and 5 mmHg to the DBP values^32^. Body weight and height were measured with the participants in light clothing without shoes. Body mass index (BMI) was calculated as body weight divided by the square of height (kg/m^2^). Blood sample was taken in the morning with at least 12 h of fasting state. All blood samples were taken on the same day. The eGFR (estimated glomerular filtration rate) was calculated according to the MDRD (Modification of Diet in Renal Disease) equation.

### Assessment of PM_10_ exposure

**Figure 3:**
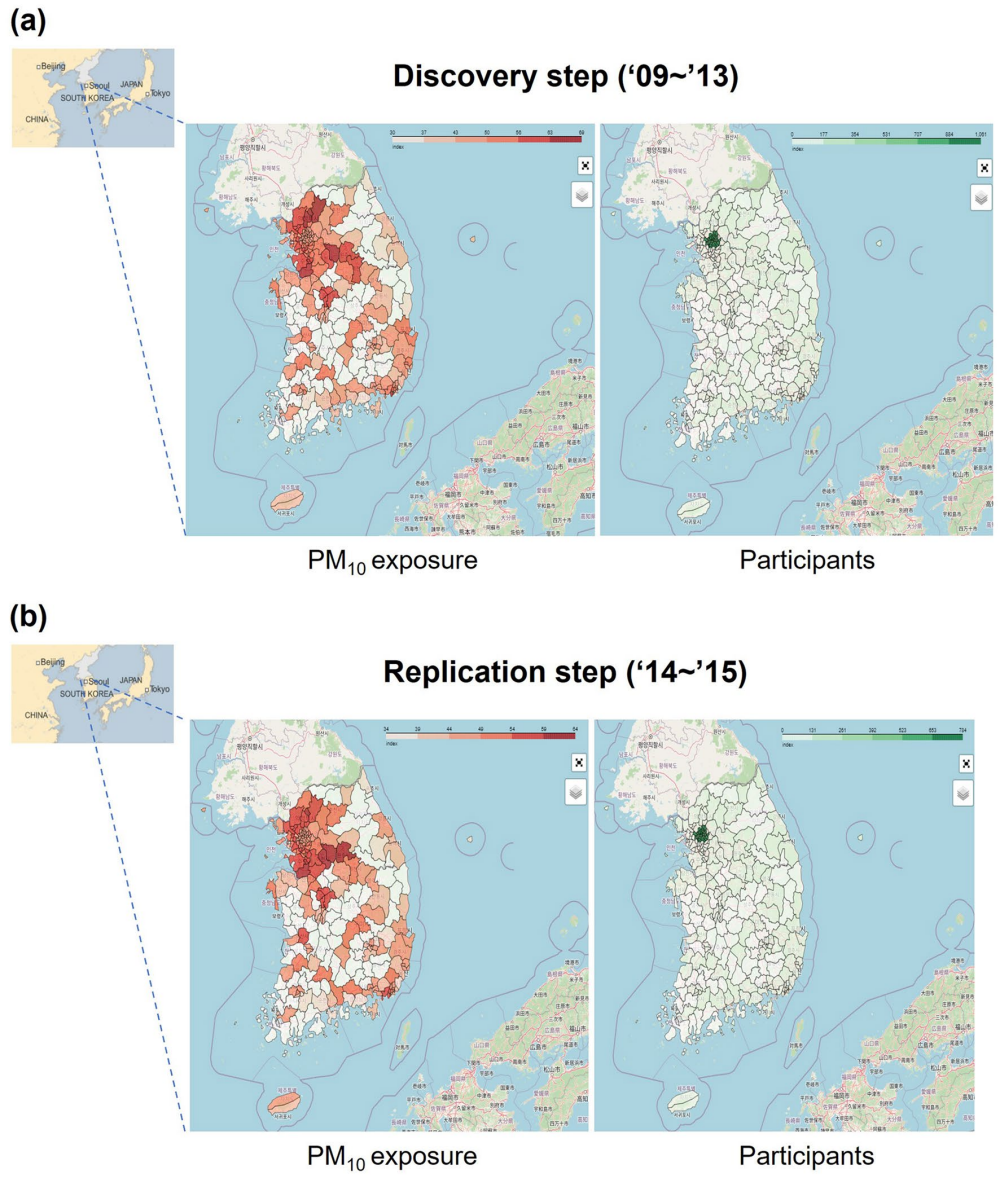
The annual average concentrations of ambient PM_10_ at each monitoring site and the distribution of the study participants in (**a**) Discovery step and (**b**) Replication step, respectively. The maps were generated by Python software, version 3.8.8 (https://www.python.org).

To estimate the exposure of participants to PM_10_, we used real-time atmospheric monitoring data from the Ministry of the Environment of Korea (https://www.airkorea.or.kr). We obtained atmospheric monitoring data for PM_10_ concentrations for every 24 h from January 1, 2009, to December 31, 2013, at approximately 300 national monitoring stations. We calculated the annual average concentrations of ambient PM_10_ at each monitoring site. We used each participant’s residential postal zip-code data and the nearest monitoring station to each participant’s residence. The maps regarding PM_10_ concentration and the distribution of the study participants were shown in Fig. 3. The maps were generated by Python software, version 3.8.8 (https://www.python.org). The PM_10_ exposure was classified into quartiles, and we used groups of those with low-to-moderate exposure (quartiles 1–3) and high exposure (quartile 4) for the genome-wide interaction analysis.

### Discovery step SNP genotyping

We extracted genomic DNA from the peripheral blood leukocytes of all participants using a QuickGene-610L device (Fujifilm, Tokyo, Japan) according to a standard protocol. For the discovery stage, samples were genotyped using a Human Core Bead-Chip kit (Illumina, San Diego, CA, USA). To minimize the possible genotyping errors, the single nucleotide polymorphisms (SNPs) were excluded by the criteria defined by Hardy–Weinberg equilibrium (*p* < 0.0005), with a call rate (< 99%) and the minor allele frequencies < 1%. After evaluating quality, a total of 196,963 SNPs were used in our GWIS.

### Replication step SNP selection and genotyping

To validate the candidate loci in the discovery step, we conducted a replication study using independent samples. We used 1281 participants enrolled from 2014 to 2015 at the same Health Promotion Center of Seoul National University Hospital in a replication analysis. The phenotypic information and genomic DNA were collected in the same manner as described for the discovery step. We selected 11 independent SNPs for the replication test considering their linkage disequilibrium (LD) relationship and the distance with nearest genes. Among the top 10 SNPs for SBP, rs1555454 exhibited a very strong LD relationship (*r*^2^ = 0.99) with the rs2273717 of the *TEK* gene, and therefore, one (rs2273717) of the two variants was selected for a replication. Three SNPs (rs1795849, rs10861229, and rs10778338) located in the intron of the *CHST11* gene were also in the strong LD relationship (*r*^2^ > 0.8), of which SNP rs1795849 was finally included in the replication phase. In addition, SNP rs13402716 was not selected based on priority of gene distance (more than 100kB away from the nearest genes). In case of DBP, the SNP rs1882492 was excluded for a replication because it showed a strong LD relationship (*r*^2^ = 0.82) with the rs1795849 in the *CHST11*. The three SNP (rs8033729, rs2965318, and rs841470) was not selected based on priority of gene distance (more than 100kB away from the nearest genes). In addition, rs1235304 was initially included for the replication, but it was excluded due to the failure to produce TaqMan probe. Therefore, among the top 10 suggestive SNPs from each genome-wide interaction analysis, we finally included six representative SNPs for SBP and five SNPs for DBP. The SNPs selected were genotyped using a TaqMan SNP Genotyping Assay (Applied Biosystems, Carlsbad, CA, USA) with a ViiA7 instrument at the Genomic Medicine Institute Research Service Center.

### Statistical analysis

We conducted a genome-wide SNP by PM_10_ exposure interaction study for BP-related traits, such as SBP and DBP, in Korean adults. SNPs were tested using a multiple linear regression method via additive genetic models using PLINK software, version 1.9 (http://pngu.mgh.harvard.edu/Bpurcell/plink/). The distribution of BP traits did not follow a normal distribution (Kolmogorov–Smirnov, *p* < 0.05), and so we applied a log transformation to SBP and a square root transformation to DBP. The results of the discovery stage were adjusted for site of recruitment, BMI, and age. A regional plot was created using LocusZoom software (http://locuszoom.sph.umich.edu/locuszoom/). In a replication step, similar to the discovery step, we performed multiple linear regression analyses to determine the candidate SNPs for the interaction with exposure to PM_10_ for BP traits with the adjustment for BMI, sex, and age. In addition, we used the t-test to identify the difference in BP level between low to moderate and high exposure groups according to each genotype of the suggestive SNP. A threshold *p* of 0.05 was used to assess the significance of replication. IBM SPSS Statistics for Windows, version 25 (IBM Corp., Armonk, NY, USA) was used for the statistical analyses.

## Data Availability

The datasets generated and/or analyzed during the current study are deposited in K-BDS (Korea BioData Station, https://kbds.re.kr) with the accession ID (PRJKA507860).
